# Navigating global socialism: Tanzanian students in and beyond East Germany

**DOI:** 10.1080/14682745.2018.1485146

**Published:** 2018-06-25

**Authors:** Eric Burton

**Affiliations:** a Research Project ‘Socialism Goes Global’, Department of History, University of Exeter, UK; b Department of African Studies, University of Vienna, Vienna, Exeter

**Keywords:** Tanzania, East Germany, students, socialism, mobility

## Abstract

This article investigates tensions and dynamics in global socialism through a focus on Tanzanian students in East Germany between the late 1950s and 1990. Disciplinary techniques partially known from Tanzania and everyday strategies of survival explain why most students complied with official requirements, but did not necessarily agree with East German ideological tenets. Additionally, throughout the decades, mobility across the Iron Curtain remained an important strategy to further own interests. The article concludes that an analytical framework spanning several decades and paying attention to dynamics in the country of origin sheds new light on agency and mobility among ‘East’, ‘West’, and ‘South’ during the Cold War.

## Introduction: students and the global struggle for ideological hegemony

Socialism has never been a unified force. Nobody was in a better position to experience the consequences of ideological divergences within the socialist world than persons who travelled and navigated between different socialisms, including students from socialist countries in the global South who attended East European universities during the Cold War period. A Tanzanian who went to study engineering in the German Democratic Republic (GDR) in 1975 found himself at the intersections of the cooperation-cum-competition of varying models of state socialism. Coming from a country that – from the vantage point of GDR authorities – promoted a heterodox view of socialism, he remembered that Tanzanians were welcomed with a large dose of suspicion:

Tanzania was socialist, but it was more aligned to China, to Mao Tse-tung. And that’s why we were not recognised there [in the GDR], because we were not in the line of Marxism.^1^1Interview #75, Tanzanian student in GDR (Dipl. Ing.) and Federal Republic of Germany (FRG) (PhD).

This memory resonates with recent scholarship which has highlighted that the Cold War’s constellations were much more complicated than the simple dichotomies of East vs. West or capitalism vs. socialism, as several players within the global socialist camp competed for influence and ideological leadership, while liberation movements and progressive government in postcolonial countries pushed for further decolonisation.^2^2Gregg A. Brazinsky, *Winning the Third World*: *Sino-American Rivalry during the Cold War* (Chapel Hill: University of North Carolina Press, 2017); Jeffrey James Byrne, *Mecca of Revolution. Algeria, Decolonization, and the Third World Order* (New York: Oxford University Press, 2016); Jeremy Scott Friedman, *Shadow Cold War: The Sino-Soviet Competition for the Third World* (Chapel Hill: University of North Carolina Press, 2015); Leslie James and Elisabeth Leake, eds., *Decolonization and the Cold War: Negotiating Independence* (London: Bloomsbury, 2015); Tony Smith, ‘New Bottles for New Wine: A Pericentric Framework for the Study of the Cold War,’ *Diplomatic History* 24, no. 4 (2000): 567–91. The post-World War II divergence of state-socialist models put Soviet affiliates like the GDR into the position of having to ‘defend’ Marxism-Leninism not only against capitalism and social democracy to its ‘right’, but also against Maoism and other socialisms to its ‘left’.^3^3Quinn Slobodian, ‘The Maoist Enemy: China’s Challenge in 1960s East Germany,’ *Journal of Contemporary History* 51, no. 3 (2016): 635–59.

Indeed, there were reasons to interpret Tanzania’s version of African Socialism – *ujamaa*, literally ‘familyhood’ – as belonging to the Chinese camp. Although *ujamaa* was no derivative of Maoism, there were significant overlaps. Tanzanian president Julius Nyerere, who was *ujamaa’s* main architect, combined a socialist, but explicitly non-Marxist, stance and non-aligned international orientation with the embrace of socioeconomic egalitarianism, ‘self-reliance’, rural collectivism, and state ownership of key industries.^4^4Priya Lal, *African Socialism in Postcolonial Tanzania: Between the Village and the World* (Cambridge: Cambridge University Press, 2015); Leander Schneider, *Government of Development. Peasants and Politicians in Postcolonial Tanzania* (Bloomington: Indiana University Press, 2014). By the late 1960s, China had become one of Tanzania’s closest allies and one of the most important donors.^5^5Alicia Altorfer-Ong, ‘Old Comrades and New Brothers: A Historical Re-Examination of the Sino-Zanzibari and Sino-Tanzanian Bilateral Relationships in the 1960s’ (Phd diss., LSE, 2014); Priya Lal, ‘Maoism in Tanzania: Material Connections and Shared Imaginaries,’ in *Mao’s Little Red Book: A Global History*, ed. Alexander C. Cook (Cambridge: Cambridge University Press, 2014), 96–116; Jamie Monson, *Africa’s Freedom Railway. How a Chinese Development Project Changed Lives and Livelihoods in Tanzania* (Bloomington: Indiana University Press, 2009). What China did not offer was, however, larger numbers of scholarships for higher education.

Yet, without qualified personnel to man the institutions of the newly independent state and positions in the economy, development prospects would remain dim so that Tanzania, like many other African countries, sent young citizens wherever possibilities for academic training opened up, no matter if ‘East’, ‘West’, or ‘South’ – although Western degrees continued to enjoy the greatest prestige.^6^6Eric Burton, ‘African Manpower Development during the Global Cold War. The Case of Tanzanian Students in the Two German States,’ in *Africa Research in Austria. Approaches and Perspectives*, eds. Andreas Exenberger and Ulrich Pallua (Innsbruck: Innsbruck University Press, 2016), 101–34; Constantin Katsakioris, ‘Soviet Lessons for Arab Modernization: Soviet Educational Aid to Arab Countries after 1956,’ *Journal of Modern European History* 8, no. 1 (2010): 85–106. The majority of Africans who studied in Eastern Europe – including Tanzanians in the GDR – qualified in technical and science subjects for which virtually all African states had the largest requirements, rather than in the arts and social sciences. They were expected to return as qualified, but docile, citizens ready to invest their skills in the development of the nation. Yet the overarching interests of governments and sending institutions could never fully determine the trajectories, experiences, and decisions of students that went to study on both sides of the Iron Curtain from the late 1950s until 1990.

Quite the opposite, as an evolving body of literature about ‘Third World students’ has shown, sending and receiving countries were themselves affected and shaped by these exchanges and circulations of persons who were crossing political, ideological, economic, and cultural boundaries.^7^7Quinn Slobodian, ed., *Comrades of Color. East Germany in the Cold War World* (New York: Berghahn, 2015). Rather than being ‘brainwashed’ during their stay, students from Africa, Asia, and Latin America were important catalysts of student activism and vocal advocates of their own rights.^8^8Young-Sun Hong, *Cold War Germany, the Third World, and the Global Humanitarian Regime* (New York: Cambridge University Press, 2015); Quinn Slobodian, ‘Bandung in Divided Germany. Managing Non-Aligned Politics in East and West,’ 1955–63, *The Journal of Imperial and Commonwealth History* 41, no. 4 (2013): 644–62. Confronting the homogenising political rituals of state socialism, they ‘probed the limits of internationalism’ as understood and sanctioned by East European authorities.^9^9Maxim Matusevich, ‘Probing the Limits of Internationalism: African Students Confront Soviet Ritual,’ *Anthropology of East Europe Review* 27, no. 2 (2009): 19–39; Julie Hessler, ‘Death of an African Student in Moscow. Race, Politics, and the Cold War,’ *Cahiers du monde russe* 47, no. 1–2 (2006): 33–64. In this perspective, students from the global South were politicised and politicising agents who forcefully resisted ideological patronage.

This argument, foregrounding non-compliance and unruliness, sits uneasily with the finding that most students who came to Eastern Europe in the years and decades after Bandung proved to be a rather pragmatic cohort interested in qualification rather than politics and socialism as such. The vast majority of former students who talked and wrote about their experiences in the Soviet Union not only felt deep gratitude for having received formal education, but often fondly remembered their time overseas as the best of their lives.^10^10Tobias Rupprecht, ‘Gestrandetes Flaggschiff. Die Moskauer Universität der Völkerfreundschaft,’ *Osteuropa* 1 (2000): 95–114; Maxim Matusevich, ‘Journeys of Hope. African Diaspora and the Soviet Society,’ *African Diaspora* 1, no. 1 (2008): 53–85. Jocelyn Alexander and JoAnn McGregor similarly find that USSR-trained intelligence cadres from Zimbabwe valued their experience in Soviet ‘living socialism’, which for them was indeed characterised by egalitarianism, anti-racism, and the universal fulfilment of basic needs.^11^11Jocelyn Alexander and JoAnn McGregor, ‘African Soldiers in the USSR. Oral Histories of ZAPU Intelligence Cadres’ Soviet Training, 1964–1979,’ *Journal of Southern African Studies* 43, no. 1 (2017): 49–66. Yet the argument of Alexander and McGregor does not stop there. As they point out, the intelligence cadres’ oral accounts of their experiences in the Soviet Union are marked by a stark contrast to conditions in Rhodesia, then still ruled by a white minority regime. It is this very contrast that shaped the travellers’ impressions, their space for manoeuvre, and their memories.

I argue that we should extend this analytical view – which goes beyond an isolated treatment of experiences in Eastern Europe – also to students. The experiences of travellers between socialisms, and in the Cold War world at large, need to be understood in a broader framework that takes cognisance of conditions and dynamics ‘back home’ and simultaneously situates the trajectories of overseas students – which often included sojourns in, or references to, the West – within changing global tensions and inequalities.^12^12The same is true of contract workers, as shown in a nuanced article by Marcia C. Schenck, ‘From Luanda and Maputo to Berlin. Uncovering Angolan and Mozambican Migrants’ Motives to Move to the German Democratic Republic (1979–1990),’ *African Economic History* 44 (2016): 202–34. This framework, putting the East European experience into a larger temporal and spatial perspective, addresses several restrictions in the literature. First, studies on East-South cooperation in the academic realm, and student exchanges in particular, have overwhelmingly concentrated on the 1960s so that we are unable to trace shifts over longer periods of time and still know little about historical conjunctures.^13^13In addition to the works already cited, this applies to Damian Mac Con Uladh, ‘Studium bei Freunden? Ausländische Studierende in der DDR bis 1970,’ in *Ankunft, Alltag, Ausreise: Migration und interkulturelle Begegnung in der DDR-Gesellschaft*, eds. Christian T. Müller and Patrice G. Poutrus (Cologne: Böhlau, 2005): 175–220; Rossen Djagalov and Christine Evans, ‘Moskau 1960: Wie man sich eine sowjetische Freundschaft mit der Dritten Welt vorstellte,’ in *Die Sowjetunion und die Dritte Welt: UdSSR, Staatssozialismus und Antikolonialismus im Kalten Krieg 1945–1991*, ed. Andreas Hilger (Munich: Oldenbourg, 2009), 83–105; Frank Hirschinger, *Der Spionage verdächtig. Asylanten und ausländische Studenten in Sachsen-Anhalt 1945–1970* (Göttingen: V & R Unipress, 2009); Constantin Katsakioris, ‘Transfers Est-Sud. Échanges éducatifs et formation de cadres africains en Union soviètique pendant les années soixante,’ *Outre-Mers. Revue d’histoire* 94, nos. 354–355 (2007): 83–106. Second, almost all of the accounts available rely on materials from European archives and thus exhibit a source bias which leads to a neglect of the perspectives and biographies of those who came to study.^14^14As is stated by Damian Mac Con Uladh, ‘Guests of the Socialist Nation? Foreign Students and Workers in the GDR, 1949–1990’ (PhD diss, University College London 2005), 223. Only recently have scholars – more often anthropologists and sociologists, rather than historians – begun to consult archives in the sending regions, to interview former students, and to pay attention to the life trajectories before and after the educational sojourn, pointing out the difficulties in converting the experiences and cultural, social, symbolic, and political capital accumulated abroad.^15^15Monique de Saint Martin, Scarfò Grazia Ghellab, and Kamal Mellakh, eds., *Étudier à l’Est. Expériences de diplômés africains* (Paris: Karthala, 2015); Andrea Schmelz, *Bildungsmigranten aus Afrika und Asien. Interkulturalität, Umbrüche und Neuorientierungen im geteilten und wiedervereinigten Deutschland* (Frankfurt am Main: IKO, 2004); Svetlana Boltovskaja, *Bildungsmigranten aus dem subsaharischen Afrika in Moskau und St. Petersburg. Selbst- und Fremdbilder* (Herbolzheim: Centaurus Verlag & Media, 2014).

This article aims to address these gaps, discussing the agency of Tanzanians who studied in East Germany between the early 1960s and the late 1980s. It pays particular attention to meaning-making and political subjectivity embedded in the life trajectories of the students, as opposed to an isolated treatment of their ‘East German experience’. In examining the agency of Tanzanians in East Germany, this study brings into dialogue archival materials from the two German states, Tanzania, and the United Kingdom with personal narratives, i.e. autobiographical writings and oral-history interviews.^16^16Between 2014 and 2017, I interviewed 19 Tanzanians (18 male, one female) who studied in East Germany between the 1960s and the 1980s, including first degree and PhD students in engineering, tropical agriculture, economy, and medicine. Eight of them went on to pursue additional academic qualifications in West Germany. Further interviews relevant here were conducted with East German academics and university functionaries, (East) German women who married Tanzanian students, as well as Tanzanians who studied in West Germany and Romania. A few of the interviews were conducted together with Atuswege Burton or Berthold Unfried. Most of the interviews have been anonymised as some informants expressed security concerns, hinting at similarities (and legacies) between contemporary Tanzania and East Germany in matters of surveillance and freedom of speech. I treat these personal narratives here as indicative (though not necessarily representative) of broader social experiences, the examination of which illuminates both particular social positions and broader historical processes.^17^17Mary Jo Maynes, Jennifer L. Pierce, and Barbara Laslett. *Telling Stories: The Use of Personal Narratives in the Social Sciences and History* (Ithaca: Cornell University Press, 2008), 128. While sources such as memoirs and oral histories have played an important role in countering archival silences and biases, particularly in African studies, the narratives are by no means direct ‘voices’ of the past as narratives are mediated by political agendas and more recent discourses.^18^18Luise White, Stephan F. Miescher, and David William Cohen, eds. *African Words, African Voices: Critical Practices in Oral History* (Bloomington: Indiana University Press, 2001). Memories of socialism both in Tanzania and East Germany are tainted by a general understanding that these socialisms ‘failed’, meaning that there is usually a moment of individual distancing to the socialist past. At the same time, central tenets of these socialisms are embraced and taken as a point of departure to criticise the current neoliberal era’s inequities.^19^19The Tanzanian landscape of memories of socialism is examined in Marie-Aude Fouéré, ed. *Remembering Julius Nyerere in Tanzania: History, Memory, Legacy* (Dar es Salaam: Mkuki na Nyota, 2015). Given that returnees from Eastern European universities were sometimes looked upon with suspicion and their degrees had less symbolic value as compared to Western credentials, many interviewees were also eager to highlight the ‘non-ideological’ character of their education in the GDR, thereby asserting their own qualification, knowledge, and professionalism. Still, ideological questions and political agency played a prominent role in these narratives. With few exceptions, archival evidence and oral-history narratives point to a sense of restricted political agency among Tanzanian students, as told through instances of disciplinary action and stifled activism.^20^20While I understand agency in general as an individual’s capability to control and shape the social relations he or she is part of by making use of material and cultural resources, political agency is here understood as the socially shaped capacity to engage, individually or collectively, with and have an impact upon matters that were subjectively (i.e. from the perspective of the actors involved) conceived of as political. Cf. Maynes et al., *Telling Stories*, 26–30. Students navigated between expectations of allegiance to models of socialism in both the GDR and Tanzania, often informed by further border crossings and personal encounters with capitalism. Following a short overview of the changing conditions of overseas studies, informed by state motives, this article goes on to discuss different shades of political agency. In the final two sections, it shows how political agency and subjectivity were informed by earlier experiences in socialist Tanzania as well as the possibility of crossing the Iron Curtain.

## Routes, bursaries, and the imperatives of national development

In the 1950s and early 1960s, South-East travels were shaped to a large extent by individual agency as a variety of trade unions, political parties, and other non-state organisations were involved in sending and receiving students and some even came on their own accord. Colonial governance was unable to regulate these flows. From the late 1950s onwards, British intelligence reports from East Africawarned about an increasing ‘movement of students to communist countries’.^21^21In 1964, the governments of Tanganyika and Zanzibar united to become the United Republic of Tanzania, within which Zanzibar continued to enjoy substantial autonomy. This was seen as a flow that ‘no security measures alone [were] likely to interrupt’.^22^22Commissioner of Police to Permanent Secretary, Ministry of Security and Immigration, 6 February 1960, FCO 141/17830, United Kingdom National Archives, Kew (UKNA). I would like to thank George Roberts for bringing this file to my attention. In the postcolonial era, migration came under the auspices of ‘manpower development’. The regime of independent Tanganyika, just like the nationalist movement’s leadership in the run-up to independence, was pragmatic in sourcing scholarships from any possible country and institution.^23^23Burton, ‘African Manpower Development.’ Given the extent to which the British had neglected higher education in the territory – there were hardly any Africans in key professions, with 16 doctors, one engineer, and two lawyers present in the territory in 1961 – the postcolonial government pushed aside anti-communist concerns.^24^24John Iliffe, *A Modern History of Tanganyika* (Cambridge: Cambridge University Press, 1979), 573. As educational facilities were being established and expanded within the country, citizens were sent to countries East, West, and South. According to official manpower development reports, between 900 and 1500 Tanzanians were studying abroad on state-sponsored scholarships (from both Tanzanian and foreign sources) at any point in time between 1970 and the mid-1980s.^25^25URT, *Annual Manpower Development Report to the President* (Dar es Salaam: Government Printer, 1985), 21.

The GDR had begun attracting students from colonial and ex-colonial territories in the late 1950s in a bid to promote the global advance of socialism and decolonisation. East Germany’s foreign-policy objectives, first and foremost breaking through the diplomatic isolation resulting from West Germany’s *Hallstein Doctrine*, provided additional motives to establish bonds with proto-elites, future decision-makers, and functionaries from Africa by providing full-tuition scholarships to the decolonising world.^26^26William Glenn Gray, *Germany’s Cold War. The Global Campaign to Isolate East Germany, 1949–1969* (Chapel Hill: University of North Carolina Press, 2003); Werner Kilian, *Die Hallstein-Doktrin. Der diplomatische Krieg zwischen der BRD und der DDR 1955–1973. Aus den Akten der beiden deutschen Außenministerien* (Berlin: Duncker und Humblot, 2001). While the first students from East Africa trickled into East Germany from the late 1950s, larger numbers arrived after the revolution in Zanzibar in 1964, which attracted East Germany’s attention. After the union of Tanganyika and Zanzibar to become the United Republic of Tanzania in the same year, the GDR continued to focus on Tanzania and hoped that revolution and socialism – in its Marxist-Leninist variety – would spread from Zanzibar to the mainland.

The number of Tanzanian university students in East Germany grew as hopes of diplomatic recognition persisted. It peaked with 95 students in the early 1970s, at a time when Willy Brandt’s *Ostpolitik* paved the way for ending East Germany’s international isolation.^27^27See Burton, ‘African Manpower Development,’ 65–6. According to Thomas Burgess, there were 123 Zanzibari students in East Germany in 1966. This number most likely includes other non-academic training programmes and internships as well. G. Thomas Burgess, ‘A Socialist Diaspora: Ali Sultan Issa, the Soviet Union and the Zanzibari Revolution,’ in *Africa in Russia, Russia in Africa: Three Centuries of Encounters*, ed. Maxim Matusevich (Trenton, NJ: Africa World Press, 2007), 281. At the time, Tanzanians constituted the largest group of students from sub-Saharan Africa in East Germany, later to be overtaken by an intake from the post-1977 focus countries Mozambique, Angola, and Ethiopia, whose government embraced scientific socialism and were thus closer to the GDR’s ideological stance than Tanzania. At the same time, East Germany was trying to commercialise the courses offered for foreign students (*Ausländerstudium*) in order to mitigate a balance of payments crisis and devising new strategies to access hard currency. This also meant that commercial industrial projects, like the establishment of a textile mill in the southern Tanzanian region of Mbeya, maintained the flow of Tanzanian students and trainees to the GDR, balancing the decreasing numbers of students on stand-alone scholarships financed by East Germany. In 1988, there were still 67 Tanzanian students in the GDR.^28^28‘Statistik’ fol. 100, HA II 28716, MfS, Der Bundesbeauftragte für die Unterlagen des Staatsicherheitsdienstes der ehemaligen DDR, Berlin (BStU).

## Negotiating dissent and compliance: disciplinary techniques and everyday strategies of survival

Across the colonial-postcolonial divide, the majority of students were largely indifferent to ideological struggles as an end in itself. They considered overseas studies first and foremost as a vehicle of social mobility. At the same time, however, most were eager to learn more about both capitalist and socialist varieties of modernity to draw informed lessons for one’s country, but also global struggles.^29^29See, for instance, Constantin Katsakioris, ‘Les promotions de la décolonisation. Les premiers étudiants africains en URSS et leurs désillusions, 1960–1965,’ in *Étudier à l’Est. Expériences de diplômés africains*, eds. Monique de Saint Martin, Scarfò Grazia Ghellab, and Kamal Mellakh (Paris: Karthala, 2015), 80. This is where the competition of socialisms was most obvious. Like other students from African, Asian, and Latin American countries, many Tanzanian students in the GDR defended non-Marxist concepts of African Socialism or appropriated Chinese and Cuban theories of militancy which demanded the support of armed liberation struggles and criticised the Soviet Union for its ‘revisionist’ policy of peaceful coexistence with Western imperialist powers. Maoist calls for direct confrontation with the West to speed up decolonisation resonated with anti-colonial sentiments and widespread suspicion of US-Soviet superpower competition in Africa.^30^30Friedman, *Shadow Cold War.* GDR authorities routinely dismissed such non-orthodox positions as confused, immature, and misguided, trying to defuse and suppress any curiosity that might consolidate ideological ‘confusion’.^31^31P. Terzopoulos, Einschätzung des Studenten O. G. C., 5 January 1968, fol. 7, StuA 116549, Universitätsarchiv Leipzig (UAL); Beurteilung des Studenten K., 27 November 1963, Bl. 28, StuA 12291, UAL; Einschätzung des Herder-Instituts über Y. A. H., Leipzig, 6 June 1964, fol. 43, StuA 117206, UAL. While disagreements relating to the Sino-Soviet split were frequent in the 1960s and early 1970s, struggles over the importance of armed struggle, ‘Soviet revisionism’, and Maoist ‘ultra-leftism’ only flared up sporadically in the 1980s.^32^32IM ‘Thoralf Winter,’ Bericht, Magdeburg, 25 January 1982, fol. 56, HA II/28716, MfS, BStU.

Interwoven with diverging opinions on how to tackle global issues were debates on how to build a socialist society, provoked by concrete observations and living conditions in East Germany: the state’s secular *rite de passage* for youths to join socialist society, the *Jugendweihe*, was interpreted by a group of Tanzanians as evidence for the undemocratic, anti-religious nature of the GDR and coercion applied to young citizens, while an increase in living costs (in particular, the rent of the student hostel) was denounced, in a boomeranging appropriation of Marxist terminology, as ‘exploitation’.^33^33Mac Con Uladh, ‘Guests of the Socialist Nation,’ 57. Such remarks concerned East German authorities, but they must be read against the general impression from interviews and archives that most Tanzanian students refrained from open confrontations. Although most interviewees voiced criticism of certain aspects of everyday life in the GDR – including racism, the supervision of private life, restricted movement of GDR citizens and fellow students, or missing freedom of speech – none of them condemned the GDR (or socialism) wholesale. From the interview narratives it seems that these negative experiences did not outweigh the benefits and privileges of their scholarship. The mentioned strategies for dealing with challenges, problems, and restrictions were very concrete: nodding without agreeing; employing humour in dealing with racist prejudice and exoticist stereotypes; knitting networks composed of trusted people; moving in groups to be able to defend oneself in bar brawls and fights about ‘girls’; and avoiding certain spaces (e.g. ‘workers’ quarters’) where one expected verbal abuse, provocations, and threats. Outspoken dissent and political activism, was, however, rare – especially after the early 1970s. This should, however, not be taken for uncritical acceptance or submission.

Disciplinary techniques played a large role in bringing about compliance among most foreign students, though always with a view on East German society. GDR authorities feared that the influx of unwanted ideas could potentially also destabilise the regime’s hold on its population. As the Stasi observed in the mid-1970s, ‘negative effects’ of foreign students’ presence in East Germany derived first and foremost from them being ‘carriers and propagators of hostile ideological concepts’.^34^34Probleme der politisch-operativen Sicherung ausländischer Studierender aus nichtsozialistischen Staaten in der DDR, n.p., n.d. [ca. 1974], fol. 15, HA XX/3683, MfS, BStU. Thus, the GDR put in place mechanisms to reward allegiance and punish, albeit often only indirectly and comparably mild, dissent among foreign students. The complexity of disciplining mechanisms and corresponding strategies to circumvent or come to terms with official expectations can be better understood with a resort to typologies of compliance. In classic social- and political-science accounts, compliance has been conceptualised as being based on coercion (force or the threat of force), utilitarian reasoning (the calculation of gains and losses for the individual or group), or consensus (the sharing of values and outcomes).^35^35Lisa Wedeen, *Ambiguities of Domination. Politics, Rhetoric, and Symbols in Contemporary Syria* (Chicago: University of Chicago Press, 1999), 144–6. Wedeen also proposes disciplinary-symbolic power as a fourth category of compliance, which I omit here. These different forms of compliance were, however, interrelated.

Official material incentives to study hard, for instance, had two sides. On the one hand, a scholarship for excellent academic results (and taking part in political activities) provided a source of additional income, but on the other, it could be received as a subtle threat of potential punishment as well. A student of textile engineering who received an additional scholarship for excellent performance remembered that it might have been discontinued anytime, which led her to avoid conflicts (*ugomvi*) with the authorities and abstain from criticism of the GDR.^36^36Interview #76, Tanzanian student in the GDR. Chances are that she might not have considered dissent as an option anyway, with or without the award, given that she did not mention any major frustrations – this is impossible to ascertain. What is interesting, however, is that it is difficult to put her account into the box of utilitarian, coercive, or normative compliance.^37^37Wedeen, *Ambiguities of Domination*, 144–5. In her narrative, it was the award of the scholarship which opened up a new possibility for punishment (withdrawal of the scholarship) so that compliance could be achieved without even uttering a threat. The fear of being punished sufficed. In a way, the scholarship paved the way for coercive compliance. Yet, this only worked because of the utilitarian reasoning, i.e. the connection with material gains.^38^38In general, students who failed their studies were exmatriculated and sent home; in the case of this female student, however, this ‘threat’ was insignificant because she performed well. Additionally, she also mentioned that conforming to Marxist-Leninist orthodoxies came easily to her as she was used to a country’s efforts to promote its national ideology from Tanzania – an aspect that points to personal experiences of political subjectivation to which I will return below.

Another part of disciplinary measures in the Eastern Bloc was the establishment of streamlined national students’ unions. The associations had to be politically docile and conform to the rules of East German socialism. Their constitutions were required to be a standard format and all meetings had to be registered with the authorities. East German state security often gathered intelligence about the proceedings.^39^39HA XX/8, Information über geplante Veranstaltung im Verantwortungsbereich, Berlin, 29 December 1981, HA XX 16587, MfS, BStU. Only in some cases, including Iraq and Syria, were there irreconcilable divisions between communists and adherents of other socialism, so that there were no unified associations.^40^40Mac Con Uladh, ‘Guests,’ 59–60. In other cases, nominal unity concealed political divisions. The association of Tanzanian students in the GDR largely operated according to the expectations and guidelines of East German authorities (at least from the early 1970s onwards, which is the time for which records are available). Its leaders saw the main purpose of the association in ensuring progress in courses and upholding a sense of patriotism and duty to return, not in activism. As a former representative of the Tanzanian student union remarked, that was thought of as impossible anyway: ‘East Germany wouldn’t have allowed [us] to engage in politics; that was forbidden.’^41^41Interview #65, Tanzanian student in the GDR: ‘East Germany wasingeruhusu kushughulika na politics hiyo, ilikuwa ni marufuku.’ The disciplinary function of the national association, directed at its members, did impact the understanding of social roles, although the overall impact seems to have been limited. In the interviews, former leaders of the union emphasised their role in helping underperforming students and calling to order those who valued nightlife over classroom activities. In speeches, they called for the support of *ujamaa*, but also encouraged their fellow Tanzanians to study Marxism-Leninism eagerly and take part in activities organised by GDR organisations.^42^42Maganya, ‘18 Jahre Tansania,’ Speech manuscript, 1982, fol. 36–40, DIB 346, UAL. This latter was particularly the case in the early 1980s, when the old guard of Tanzania’s ruling party turned towards Eastern Europe and flirted with the concept of Leninist vanguardism as Western donors exerted political and financial pressure to implement macroeconomic reforms. Apparently, an ideological double identification with *ujamaa* (as far as Tanzania was concerned) and Marxism-Leninism (as far as the GDR was concerned) was the pathway that could be trodden most easily in this social position.

Interviewees who had not held any function in the association remembered that the union’s role was, in fact, rather limited and mostly confined to the organisation of parties. Archival evidence supports both impressions: the willingness of student leaders to discipline others as well as the fact that the bulk of activity related to gatherings and social events.^43^43General Secretary of Tanzanian Students Union to Dpmt. ‘Ausländerstudium’ of the Karl-Marx-Universität Leipzig (KMU), 3 March 1971, DIB 346, UAL. On the desired disciplining function of the unions, see ‘Zu Ergebnissen und Problemen im Ausländerstudium 1975/76,’ n.d., p. 10, DR 3/2. Schicht/4058, Bundesarchiv Berlin-Lichterfelde (BArch Berlin). Whereas other unions, like those of Nigeria or Ethiopia, were at times deeply divided over ideological, political, and ethnic cleavages, the remarkable success of the nation-building project in Tanzania and the absence of major ethnic rivalries and political conflicts meant that the association of Tanzanian students in the GDR did not have to deal with major internal conflicts. During the 1970s, West German and East German observers agreed that in contrast to other African groups, Tanzanian students proved to be exceptionally patriotic as they acknowledged both their privilege to belong to the ‘happy few’ and their responsibility to return and develop their country, leading to an almost universal readiness to return without further incentives needed.^44^44‘Grundsatzüberlegungen zur Reintegration der in der BRD aus- und fortgebildeten Staatsangehörigen von Entwicklungsländern,’ Bonn-Bad Godesberg, 7 November 1972, p. 3, B 138/34571, Bundesarchiv Koblenz (BArch Koblenz); ‘Politisch-ideologische Situation in der Union der tansanischen Studenten in der DDR,’ 14 March 1977, HA XX 3210, MfS, BStU. The persistence of the *ujamaa* discourse abroad shows how the ideology of the socialist one-party state had permeated Tanzanian society, or at least resonated among its educated members.

Written and oral testimonies on these aspects offer different perspectives, but once we think about compliance (rather than either submission or resistance), these are not totally irreconcilable. In an interview in 2014, the former president of the Tanzanian student association described himself as entirely apolitical, due to his faith, as he belonged to the Jehovah’s Witnesses.^45^45Interview #65, Tanzanian student in the GDR. From this vantage point, an affirmation of any political or official function was thus unlikely. Although he emphasised not being particularly interested in politics even earlier in his life, saying that other people had pushed him into this leadership position, it is possible that his position then had not been as distant as it was in 2014. A Stasi report penned almost four decades earlier had lauded his ‘firm leadership style’ (*straffe Leitungsmethode*) and exceptional ability to enforce discipline among Tanzanian students. Thanks mainly to his leadership, the report noted, many Tanzanians took part in study circles – despite their religious sentiments. (In fact, GDR authorities also took care not to offend religious feelings; in 1977 they even provided a PhD scholarship for a Tanzanian who had studied theology at the Pontifical University in Rome, arguing he had a ‘progressive worldview’.)^46^46Oelschlägel to Gomille, Dar es Salaam, 18 March 1976, HA XX 3210, MfS, BStU. According to the Stasi, the Maoist influence had been very strong among Tanzanians but had been ‘principally abolished’ by 1974.^47^47‘Politisch-ideologische Situation in der Union der tansanischen Studenten in der DDR,’ 14 March 1977, HA XX 3210, MfS, BStU. The testimony of another GDR graduate pulls in a different direction and suggests that the perception of global power shifts rather than ideological patronising were responsible for this change. He argued that everybody at the time thought that the world was inevitably moving towards socialism. Given the success of the Marxist-inspired liberation movements in Portuguese Africa, the Ethiopian revolution, and the US retreat from Vietnam in 1974/1975, the path of history seemed to be leading towards the predominance of a Soviet-dominated socialist world system.^48^48Interview #75, Tanzanian student in GDR (Dipl. Ing.) and FRG (PhD). The significance of the Chinese challenge waned considerably as the Soviet Union adopted Chinese positions on the armed struggle and China itself turned inward during the post-Maoist reform period.^49^49Friedman, *Shadow Cold War*. This narrative of an ‘objective’ appeal of socialism also serves to rationalise, rather than condemn or silence, any past sympathies for state socialism, sympathies which otherwise might be hard to communicate in the neoliberal era.

Apart from the student union, there were other disciplining mechanisms. GDR authorities actively devalued alternative opinions and ideological stances outside the university as well. They tried to control ‘ideological trafficking’, as in the case of a Zanzibari student who, in the early 1960s, had to hand over to the East German authorities a book he had just acquired from the Albanian embassy.^50^50Interview with Adam Shafi, Dar es Salaam, 27 March 2014. For similar incidents see Hirschinger, *Der Spionage verdächtig*, 139–40. The incident occurred after Albania had openly sided with China’s ideological positions and the Soviet Union had discontinued diplomatic relations in December 1961; Albania had also already withdrawn all students from East Germany in October.^51^51Mac con Uladh, ‘Guests,’ 47. Like the Mao bible some years later, the book had represented a threat to the ideological hegemony that East German authorities were keen to establish. Other interviewees remembered that they were discouraged from consuming Western media and were encouraged to read the party’s newspapers instead. They considered as spies those German students who stayed in the same room and relayed information about academic performance and the private activities of foreign students to the authorities: ‘The FDJ [*Freie Deutsche Jugend*, the party’s youth organisation] was part of the Stasi system,’ a former student of veterinary medicine opined during the interview.^52^52Interview #65, Tanzanian student in the GDR: ‘FDJ pia ilikuwa system ya Stasi.’ Although this view might also have been shaped by post-1989 discourses in which the activities of the Stasi figured as chief evidence of the East German regime’s oppressive nature, complaints about this scheme of personalised care, support, and surveillance through peers can also be found in archival records. The same is true for the system of peer-observation through fellow students of the hosting state that was put in place in other COMECON countries as well.^53^53Hirschinger, *Der Spionage verdächtig*, 101. The effectiveness of this system depended on contingent factors, first and foremost the complicity and interests of both the local and foreign students involved.^54^54MHF, ‘Ergebnisse und Probleme bei der Ausbildung und Erziehung ausländischer Studierender,’ n.p., n.d. [1977], fol. 156, HA XX/3683, MfS, BStU. Some interviewees also expressed gratefulness for the support received within these relations, while others said that there had been no personal support worthy to be mentioned whatsoever.

Resistance and dissent were not absent, but GDR authorities were eager, and often successful, at channelling or addressing the concerns and thus bringing about compliance. This is illustrated by ideological classes. As in the Soviet Union, these had been optional initially and became compulsory for foreign students in 1969.^55^55Mac Con Uladh, ‘Guests,’ 58; Constantin Katsakioris, ‘Creating a Socialist Intelligentsia. Soviet Educational Aid and its Impact on Africa (1960–1991),’ *Cahiers d’Études africaines* LVII, no. 2 (2017), 269. Although this policy change was a reaction to foreign students’ supposed ‘ultra-left’ or ‘petit-bourgeois’ aberrations, it might be interpreted less as an effort to indoctrinate – authorities were well aware of the limits of such strategies – and more as a technique to partially allow, discuss, and channel dissent, especially given that students from non-socialist countries (i.e. those not represented in the COMECON) received a milder form of classes separate from students from communist ‘brother countries’. A former engineering-sciences student also related that he deliberately failed exams in *Marxismus-Leninismus* in the mid-1970s, not seeing any benefit in this course as ‘[i]t was not contributing anything to my civil engineering career’. However, due to his excellent grades in all other subjects, East German authorities became suspicious: ‘They told me intelligence does not separate the subjects.’^56^56Interview #75, Tanzanian student in GDR (Dipl. Ing.) and FRG (PhD). After a personal conversation (*Aussprache*), a favourite tool of officials to discipline individuals, he abstained from further deliberate failings and wrote what he was expected to write. That did the job. The GDR required compliance, but not belief. Acting ‘as if’ was enough.^57^57Lisa Wedeen, ‘Acting “As If”: Symbolic Politics and Social Control in Syria,’ *Comparative Studies in Society and History* 40, no. 3 (1998): 503–23. The same pattern of resisting, getting pressure from authorities and complying can also be observed in the archival record: Sektion Marxismus-Leninismus, ‘Die politisch-ideologische Arbeit mit ausländischen Studierenden im Studienjahr 1970/71,’ 14 June 1971, Technische Universität Dresden, Universitätsarchiv, DIB 602.

Although speaking one’s mind was far less risky for foreign students than for their German colleagues, many quickly realised that open dissent meant inconvenient trouble nonetheless. Several interviewees remembered instances in which they decided to ‘shut up’ (*kufunga mdomo*) in confrontation with East German officials because they feared that open dissent or inquiry could put their careers in jeopardy. The fear of being deported and returning as a ‘failure’ was very real and a considerable constraint on political agency. This extended to political rituals beyond the academic sphere as well, as he remembered: ‘We were also forced to be active politically,’ for instance by participating in the 1 May celebrations, marching past the leadership and holding up the Tanzanian flag, hailed through loudspeakers as representatives of the Tanzanian ‘proletariat’ and allies in the fights against imperialism and capitalism. ‘And you cannot tell them “That is not true!” No, we are marching (laughs). […] I was angry, but what do you do? You wanted to survive.’^58^58Interview #75, Tanzanian student in GDR (Dipl. Ing.) and FRG (PhD). As there were only few Tanzanian students where he studied, skipping the event would have made him a target of the authorities. Seeing no alternative, he decided to perform his allegiance despite his dislike for the political ritual. Compliance as a strategy of ‘survival’ seemed a wise choice also because fellow German students were just as ambivalent in their stand towards the ‘system’:

[W]e came to discover that most of the youths were against the system. We came to discover that. But they were afraid to talk. They were not talking openly against the system because that – for them – would be very dangerous. But privately, we managed to find out.^59^59Interview #75, Tanzanian student in GDR (Dipl. Ing.) and FRG (PhD).

The narrative of this engineer, who was to become a professor back in Tanzania, draws a complex picture of compliance and noncompliance, of disciplining mechanisms and own strategies to retain a space of autonomy. He observed that in public, GDR youths were acting in compliance, but without believing. They, too, were acting ‘as if’. This behaviour also depended on larger historical contexts.

## Applying Ujamaa’s lessons in East Germany

The biographical experiences and future expectations of students who came to the GDR after the mid-1960s were different from those of their predecessors in some very important aspects. In the early years, i.e. the 1950s and early 1960s, young men (and a few women) had often travelled widely even before their arrival in the GDR. In the case of disappointment or academic failure, they were optimistic about finding other opportunities if they left their initial place of study. Upon return, they were sure to profit from the policy of nationalisation in a rapidly growing bureaucracy that was experiencing its golden age.^60^60Andreas Eckert, ‘“We Must Run While Others Walk”: African Civil Servants, State Ideologies and Bureaucratic Practices in Tanzania, from the 1950s to the 1970s,’ in *States at Work: Dynamics of African Bureaucracies*, eds. Thomas Bierschenk and Jean-Pierre Olivier de Sardan (Leiden and Boston: Brill, 2014), 217. In contrast, those who were sent after the 1960s knew that their stay in East Germany might well be the only possibility of laying their hands on a degree, which was still a fairly sure entry ticket to the higher ranks of Tanzanian society. Meanwhile, however, the one-party regime had assumed a leading role in ‘manpower development’ and made use of its gatekeeper function inherited from colonial times.^61^61On the concept of the gatekeeper state see Frederick Cooper, *Africa since 1940. The Past of the Present* (Cambridge: Cambridge University Press, 2002). Narrowing down possibilities of migration and any physical movement deemed unhelpful for national development, the state also put severe restrictions on getting a passport.^62^62A personal experience of this restrictive policy is related, for instance, in Hanna Schott, *Matomora Matomora. Der längste Umweg führt nach Hause* (Schwarzenfeld: Neufeld Verlag, 2012), 66–7. Overseas training was regulated as far as possible, as it was now seen as a threat to national values and development concepts. ‘This philosophy,’ Tanzanian officials explained to visitors from abroad,

is based on the fact that local training introduces young men to the problems of this country early on in their careers and there are less chances of such Tanzanian students wishing to stay permanently in foreign countries if they go there as mature people rather than immature.^63^63UDSM, ‘A Brief for the Danish Parliamentarians,’ Dar es Salaam, 27 February 1978, FA/E90/7, Part E, Tanzania National Archives, Dar es Salaam (TNA). Confer with reference to Zanzibar: Pfannenberg, Minutes of meeting between Schneider and Said idi Bavuia, Zanzibar, 16 January 1972, DR 2/244452, BArch Berlin.

All Tanzanian university graduates who received official scholarships (both from national and foreign sources) were obliged to pay back their nation’s expenses with five years of service for the state.

Yet all of this presupposed academic success. Returning without academic credentials would have been a personal defeat with repercussions for family members and dependents as well, as the majority of Tanzanian students who went to study in the GDR came from – relatively speaking – modest backgrounds. The share of students from modest backgrounds was higher in the East, as those who could afford it still preferred studying in the West. Some interviewees explicitly described their social mobility through education as an outcome of Nyerere’s education policy which, during *ujamaa*, opened up windows of opportunity for unprivileged youth. Most of the students in East Germany could not afford regular trips home, and unlike their fellow citizens in the West, opportunities to earn hard currency for flight tickets were very limited. The lack of social capital after several years of absence from the country put graduates returning from overseas at a disadvantage in a bureaucratic environment that put a premium on having the right personal connections.

Though meritocratic and technocratic criteria were never abandoned, agreement with official policies and active participation in the nation’s socialist project of *ujamaa* became additional requirements for social mobility. After *ujamaa* had been elevated to state doctrine in 1967, mass organisations and the state’s ideological apparatuses including media and the education system aimed to produce citizens adhering to socialist and nationalist values. Those students who arrived in the GDR in the 1970s and 1980s had gathered first-hand experience of living in a one-party state and devised pragmatic strategies for dealing with its requirements of allegiance and uniformity. They knew, for instance, that unless one joined the party’s youth wing and, later, the party, avenues of upward social mobility remained closed. ‘Keeping one’s mouth shut’ (*kufunga mdomo*) was a strategy to pass unharmed through the National Service, a paramilitary work camp and political education experience which all secondary-school leavers were obliged to go through after 1966.^64^64Interview #43, Tanzanian student in GDR (Dipl.) and FRG (PhD). On the National Service, see Paul Bjerk, *Building a Peaceful Nation. Julius Nyerere and the Establishment of Sovereignty in Tanzania, 1960–1964* (Woodbridge: Boydell & Brewer, 2015), 155–80; Ronald Aminzade, *Race, Nation, and Citizenship in Post-Colonial Africa. The Case of Tanzania* (Cambridge: Cambridge University Press, 2013), 125; Godfrey Mwakikagile, *Tanzania Under Mwalimu Nyerere: Reflections on an African Statesman* (Dar es Salaam: New Africa Press, 2006), 33–4. Building a career in socialist Tanzania did not require personal political commitment, but it depended upon compliance. Acts that might be read as open dissent, contradiction of the ‘official line’, and insubordination carried risks that few were prepared to take. To be sure, the freedom to express oneself had not fully disappeared in Tanzania, as is evident in the national newspapers’ opinion pieces and letters to the editor sections. Still, a ‘spirit of fear’ had taken root which circumscribed and limited space for offering criticism and alternative thoughts.^65^65Aili Mari Tripp, *Changing the Rules. The Politics of Liberalization and the Urban Informal Economy in Tanzania* (Berkeley: University of California Press, 1997), 176.

In navigating the ideological landscape of the GDR Tanzanian students also drew on their knowledge of *ujamaa* and political education in Tanzania. After all, *Marxismus-Leninismus* classes were paralleled by political education in Tanzania as *ujamaa* was being enshrined as the one-party state’s ideology and promoted in state education and the media, especially since the late 1960s. For most, it seemed natural that each state upheld its own national ideology. The important role of the party and youth organisation was also well-known from Tanzania. Upon return (sometimes following additional stays in West Germany or other Western countries), most students re-integrated into the ideological fabric prescribed by Tanzanian authorities and avoided direct confrontation, although several returnees mentioned that they resented the intervention of party cadres (who had, very often, a significantly lower education level) into their professional work.^66^66Interview #104, Tanzanian student in GDR (Dipl. Ing.) and FRG (PhD); Interview #120, Tanzanian Student in Romania. In any case, even after 1969, the GDR did not ‘produce’ staunch Marxist-Leninists. Even those Tanzanians who had already held compatible views *before* their stay in East Germany may or may not have deepened their convictions and bonds, partly also by visiting other countries in the Eastern Bloc. Most students, however, were curious to see more than just the socialisms of Tanzania and Eastern Europe. They travelled westwards.

## Going West: Tanzanian students crossing the Iron Curtain

Germany’s location as a ‘border region of the Cold War’ allowed Tanzanian students in the GDR to travel frequently and for a wide array of purposes.^67^67Thomas Lindenberger, ‘Divided, but not Disconnected: Germany as a Border Region of the Cold War,’ in *Divided, but not Disconnected: German Experiences of the Cold War*, eds. Tobias Hochscherf et al. (New York: Berghahn Books, 2010), 11–33. In contrast to their fellow students from Africa’s Marxist-oriented ‘brother countries’ like Mozambique, Tanzanians were principally free to travel from East Germany in any direction they wished.^68^68Permission to travel depended first and foremonst on the sending country’s stance. Most African countries wanted that their citizens could move freely. For them, the ‘Iron Curtain’ was but a ‘Nylon Curtain’ that did not foreclose, but instead opened up, opportunities.^69^69György Péteri, ‘Nylon Curtain – Transnational And Transsystemic Tendencies In The Cultural Life Of State-Socialist Russia And East-Central Europe,’ in *Slavonica* 10, no. 2 (2013): 113–23.

Tanzanians crossed the border to West Germany and West Berlin – or ‘jumped the wall’ (*kuruka ukuta*), as one interviewee called it – for different ends.^70^70Interview #40, Tanzanian PhD student in FRG. Three major modes of border crossings may be distinguished. The first one was to take the *exit* option and leave the East permanently, an option taken particularly in the early 1960s. The second mode was the *zigzag* mode, i.e. repeatedly travelling westwards for shorter periods of time while one still studied in the GDR. The third mode can be seen as an *upgrade*: after finishing one’s studies in East Germany, a stay in West Germany (or another Western country) served to accumulate further symbolic, cultural, economic, and social capital that was becoming more essential as Tanzania plunged into a protracted economic crisis that affected the population well into the 1990s.^71^71I am excluding here those who stayed in Germany (or other countries in the global North) without ever returning to Tanzania. They seem to have constituted a small number, and I have not been able to interview anybody belonging to that group.

### Exit

For those who had political or other reasons to cancel their studies in the GDR, there was always the option to quit. In early 1964, the West German embassy in Moscow reported that 100 Tanganyikans who had protested against unbearable living conditions in the Soviet Union would soon be on their way to cross the Iron Curtain, heading towards the Federal Republic of Germany.^72^72Vialon to State Secretary, Bonn, 6 February 1964, p. 4, B 213/438, BArch Koblenz. The incident is very likely related to the protests and general discontentment among African students described in Hessler, ‘Death of an African Student.’ This announced number of Tanganyikans apparently never arrived; however, according to West German statistics, between 1962 and early 1964, 41 Tanganyikans had already been registered as refugees from East European countries.^73^73Twenty-one Tanganyikans had arrived from the ‘Eastern Bloc’ in 1962, eight in 1963, and 12 in 1964, which amounted to a much smaller number compared to Ghanaians, Kenyans, or Nigerians. See ‘Studenten aus Entwicklungsländern, die von Ostblockhochschulen in die Bundesrepublik Deutschland abgewandert sind,’ Stand 1.1.1964, n.p., n.d. (1964), B 213/439, BArch Koblenz. These arrivals of Tanganyikans were part of a broader current of disillusioned, or academically failing, Third World students.^74^74Slobodian, ‘Bandung,’ 654–5; Katsakioris, ‘Creating a Socialist Intelligentsia,’ 270.

While West Germany initially welcomed these so-called ‘Eastern Bloc refugees from developing countries’ and equipped many of them with further educational opportunities, this was stopped after 1964 as the costs were seen to outweigh the political gain.^75^75Minister Schröder (AA) to Minister Scheel (BMZ), Bonn, 9 April 1964, B 213/438, BArch Koblenz. West German authorities also noticed that some of the ‘refugees’ returned to East Germany (or other East European states).^76^76‘Statistik: Studienbewerber aus Entwicklungsländern, die aus der SBZ oder Ostblockländern in das Bundesgebiet kamen (1.1.-30.11.1963),’ B 213/438, BArch Koblenz. In 1966, Washington Bomani, brother of Paul Bomani who was by then Minister of Economic Affairs and Development Planning, first ‘escaped’ from the GDR to London. In London, he went to the West German embassy and applied for a scholarship, vowing to never go back to a communist country. Shortly thereafter, however, he returned to the GDR. According to his brother, the Minister, he deeply disliked West Germany *–* an explanation that was to please his East German interlocutors.^77^77Ref. IIa, Vermerk, Wiesbaden, 22 December 1964, 507/11330, Hessisches Hauptstaatsarchiv Wiesbaden (HHStAW); GDR Consul Fischer to MfAA, Dar es Salaam, 30 March 1966, Fol. 59, C 350, MfAA, Politisches Archiv des Auswärtigen Amtes, Berlin (PAAA). It seems that the ‘exit’ option was taken more rarely in the years to come because living conditions for foreign students considerably improved and selection mechanisms in sending and receiving states became stricter and more organised.

### Zigzag

Many foreign students went West via multiple short-term border crossings during weekends, holidays, and vacations. A former student of engineering sciences remembered that his own experiences on both sides of the Wall provided him with a firm base for ideological arguments:

The differences were huge. In West Berlin, we found out: That was the real life. Because in East Germany, life was very artificial. I can give you a few examples. […] If you go to a restaurant, you have to queue. You have to line up, outside, waiting to get into the restaurant. For us, it was very, very strange. You come to West Berlin, such nonsense does not exist. And […] in *Marxismus-Leninismus* subjects, we were discussing. We were trying to challenge the lecturer because of what we are seeing in West Berlin.^78^78Interview #75, Tanzanian student in GDR (Dipl. Ing.) and FRG (PhD).

After having seen ‘the West’ with his own eyes, he became more confident to challenge some of these twists and tenets in the ideological classes. The trips could thus bring about a surplus of independent personal experience that was at odds with academic lessons. But this was far from the only benefit of these westward journeys which could last from some hours (at least in Berlin) to go shopping, enjoy the nightlife, or visit friends and relatives to several weeks or months to work and gain hard currency in Scandinavian and Western European countries even without a visa. Students recognised that they could take advantage of their privilege to travel between two very different economic zones. Economic profits could be converted into prestige by means of sharing and conspicuous consumption: the latest jeans, exclusive liquor and cigarettes from West Berlin or the GDR’s hard-currency shops or the latest rock’n’roll records guaranteed a kind of avant-garde status in youth culture. Parts of the East German population also participated in the privileges of foreign students as they got access to Western clothes, music records, and beverages.^79^79For a fascinating account of the inconveniences and moral dilemmas that went along with foreigners’ privileged access to consumer goods see the letter of the South African writer Bloke Mondisane, reproduced in Slobodian, ed., *Comrades of Color*, 117–20. Engaging in such luxury was, however, at odds with the Tanzanian president’s calls for frugality: ‘If he had seen me, Nyerere would have locked me up,’ a former engineering student said as he remembered a stay in a luxury suite in Dresden’s most expensive hotel which usually only catered for socialist bureaucratic elites and Western tourists.^80^80Interview #104, Tanzanian student in GDR (Dipl. Ing.) and FRG (PhD). The ideal *ujamaa* citizen was supposed to be self-reliant and modest; overseas studies were also discouraged because elites feared that the stay abroad would awaken consumer appetites that could not possibly be saturated under Tanzanian conditions. Yet there was no way the Tanzanian state could enforce these norms. The East German appearance of Nyerere, the frugal ‘father of the nation’, was but a hovering guilty conscience in the back of one’s mind. Beginning roughly in the mid-1970s, however, there appears a new mode of socioeconomic agency in which students used the gains made from border crossings less to enjoy luxury goods and more to mitigate the effects of the economic crisis at home.

Profiting from border crossings by making use of the black-market premium in selling goods or currency was a common strategy for foreign students which took on a new meaning as the crisis in Tanzania unfolded. The economic meltdown of the 1980s had been preceded by shortages of consumer goods since the early 1970s, the oil shocks of 1973 and 1979, protracted droughts, the underperformance of the state-owned economic sector, and the retreat of the peasantry into subsistence production or informal marketing channels. Despite the policy of self-reliance, Tanzania continued to depend on cash-crop exports and donor funds to finance the household. Making a living became difficult for citizens, especially employees in the urban formal sector who, in the face of inflation and shortages, saw their purchasing power melt to a fraction of earlier years.^81^81The strategies of Tanzanians in dealing with the crisis are detailed (without mentioning international mobility) in Tripp, *Changing the Rules*; T. L. Maliyamkono and M. S. D. Bagachwa, *The Second Economy in Tanzania* (London: James Currey, 1990). It was under these circumstances that overseas scholarships became a strategy for ensuring short- and mid-term economic survival, rather than attaining educational credentials. An overseas scholarship opened economic opportunities which gave Tanzanians leverage not just in selling goods and raising their living standard in East Germany, but also in negotiating the mounting consumer-goods crisis back home. Students in East Germany started remitting hard currency acquired in the West (East German stipends, and currency in general, could not be transferred) and sending care packages with basic goods like soap and sugar to their relatives, at a time when shortages in basic goods also became more common in Eastern Europe.

### Upgrade

According to one interviewee who graduated in 1979, going west after graduating in the GDR was ‘a common thing to do to make more money’ among the Tanzanians and other foreign students he knew.^82^82Interview #63, Tanzanian student in GDR (Dipl.) and FRG (PhD). Several graduates moved on to West Germany not only to work and accumulate economic capital, but also to upgrade East German academic qualifications or wait for the East German spouse to follow.^83^83BStU, MfS, HA XX/3210, HVA Abt. III an HA XX AG Ausländer, Berlin, 14 May 1982. See also Burton, ‘African Manpower Development’; Sara Pugach, ‘African Students and the Politics of Race and Gender in the German Democratic Republic, 1957–1990,’ in *Comrades of Color: East Germany in the Cold War World*, ed. Quinn Slobodian (New York: Berghahn, 2015), 131–56. It was not unusual that those who secured a degree from an East European university would attain higher degrees and undergo further training in Western countries. Again, this strategy has gained importance due to the economic crisis in Tanzania.

With Tanzanian socialism coming under increasing pressure in the 1980s and structural adjustments under way after 1985, the state was forced to downsize. It was neither able to absorb graduates returning from overseas nor did it pay salaries that were sufficient to eke out a living. The private and parastatal sectors, which unlike most bureaucratic positions offered substantial fringe benefits, were more attractive and gained influence. Many Tanzanian students in East Germany held the view that a certificate, degree, diploma, or PhD from the West increased their chances of being employed by a ‘capitalist’ in the private sector.^84^84Wessler, Information vom 30.4.1985, Magdeburg, 2 May 1985, fol. 74, HA II/28716, MfS, BStU. The centre of gravity, not only in economic and political, but also in symbolic and cultural terms, moved westwards. Even for those who were to work, as was the rule in the decades before, for official Tanzanian institutions, the import of cars or fridges – which were, on a personal level, capital goods rather than luxury goods – was extremely helpful to kick off income-generating activities that complemented official salaries. As the ideological hegemony of *ujamaa* eroded in the course of the 1980s, battles between socialisms were rendered increasingly unimportant. In the testimonies of students who went to study in the late 1980s, such struggles are all but absent; matters of democratisation and liberalisation gain importance. The major concern for most was survival, not a grand vision of a socialist modernity.

This was even true for some of those who came directly from the political realm. A striking example is a PhD student who also served as the Tanzanian student union’s chairman in 1981.^85^85This paragraph is based on Müller, [Einschätzung des Aspiranten] B. K., Berlin, 7 May 1981, HA XX/3210, MfS, BStU; HVA Abt. III to HA XX ‘AG Ausländer,’ Berlin, 14 May 1982, ibid. Before coming to East Germany for his PhD (after a first short-term stay at a youth organisation training in 1968), he had served as a full-time party functionary on a district level for over 10 years. In direct and confidential conversations with East German officials, he demonstratively distanced himself from Maoist views and Nyerere’s non-Marxist African Socialism, arguing that Marxism-Leninism should become the party’s ideological guideline. Several East Germans, including lecturers in Dar es Salaam, testified to his ideological credentials. The chairman’s dilemma was, however, that he was forced to take a course in economics and statistics rather than in his field of specialisation, international relations. After several fruitless pleas directed at East German and Tanzanian authorities to be allowed to change, he crossed the Iron Curtain and secured a scholarship from a Christian organisation to pursue a course of his liking in West Germany. His decision to leave the GDR – symbolically significant because of his status as a representative – sent ripples through the union in East Germany. In a special meeting in 1982, the remaining leaders of the student union condemned their departed chairman for the ‘disgrace’ and ‘dirt’ he had brought upon them. The ensuing statement of the union warned against the perils of being ‘two-faced’ and was clearly meant to discourage similar defections.^86^86‘Originalkopie – Kurze schmerzliche Mitteilung des Präsidiums der TS,’ HA XX/3210, MfS, BStU. It is very likely, however, that this act of indignation was itself part of a strategy of compliance. Several members of the union were also said to be on the lookout for opportunities in the West at the very same time. The renaissance of the exit strategy occurred when the politico-ideological superstructure and the socio-economic base that informed mobility strategies were more incongruent than ever.

## Conclusion

This article has argued that, if we are to heed Quinn Slobodian’s call to engage in ‘more research on the human traffic into East Germany from […] countries of the global South’,^87^87Slobodian, ‘The Maoist Enemy,’ 658. we should strive to employ methodological approaches that yield new insights into the ideological confrontations. On sociocultural perspectives on the Cold War.^88^88Federico Romero, ‘Cold War Historiography at the Crossroads,’ *Cold War History* 14, no. 4 (2014), 685–703. On the concept of the global Cold War, see Odd Arne Westad, *The Global Cold War. Third World Interventions and the Making of our Times* (Cambridge: Cambridge University Press, 2007). Following the trajectories of Tanzanian students in East Germany, it has demonstrated the value of a transnational perspective that not only pays close attention to the experiences overseas, but articulates these experiences with opportunity structures and dynamics in the country of origin as well. This is particularly important as the relationships between these travellers and their sending and host countries were not static, but changing and dynamic. Beyond the bilateral sending country-receiving country framework, the experiences of students were also influenced by power constellations and ideological struggles on a global scale, including rivalries between competing socialisms in the heated debates of the 1960s and the renaissance of ‘scientific socialism’ in the global mid-1970s. Tracing the experiences over three decades, it becomes evident that the competition between differing models of socialism lost importance, especially as *ujamaa* had plunged into crisis and China had retreated from the Sino-Soviet battle for leadership in the socialist camp.

Most Tanzanians who studied in East Germany conceived of their stay first and foremost pragmatically as a stepping stone for their further career. Their compliance was, to a large part, a utilitarian kind of compliance that was bolstered by coercive measures and did not equal embrace of the GDR’s ideological conventions and concrete conditions – nor did a critical stance towards East German ‘real socialism’ necessitate a full rejection of Marxism-Leninism. Even those who mentioned instances of coercion shared basic tenets of the socialist project in its East German, Tanzanian, and global dimensions. Tanzanian students’ compliance with ideological demands and political rituals arose from a variety of mechanisms that involved both compulsion (more often in subtle threats rather than overt coercion) and utilitarian thinking directed at individual upward mobility. In many instances, agreement with the aims and (somewhat less frequently) methods of East German or Tanzanian socialism also played a role. This is precisely why it is so important to relate the experiences of students with contexts in their sending countries and broader historical conjunctures: both dissent and compliance – the latter not to be mistaken for obedience and uncritical acceptance – can be more fully understood.

In line with Paul Kibiwott Kurgat’s study on Kenyans students in the Soviet Union, this article has shown that a stay in East Germany did not lead to anything like a predictable ideological transformation.^89^89Paul Kibiwott Kurgat, ‘Education as a Foreign Policy Tool: Kenyan Students’ Airlifts to the Union of Soviet Socialist Republics and Eastern Europe, 1954–1991’ (PhD diss., Moi University, Eldoret, 2013). Some came with high expectations of communism and left deeply disillusioned, others held on to their sympathetic attitude towards socialism, while another – and probably the largest – group saw both light and shadow, generally highly valued the time in the GDR, and held a differentiated picture of the ideological options available. This applies, in particular, to those who used their freedom to travel. Tanzanian students had the chance to experience different political systems and could resist demonising narratives about East European communism and – in the case of those who had ‘jumped the wall’ – Western capitalism with their own, nuanced observations. This strategy of westward mobility gained importance as the economic crisis in Tanzania unfolded. With economic motives becoming more significant on both state and individual levels, there occurred a tectonic shift from grand political visions to pragmatic survival strategies to make ends meet. When the economic crisis spilled over into the social and political realm, Tanzania’s socialist experiment was increasingly undermined in the 1980s and finally dismantled in the early 1990s. Between 1989 and 1991, socialist states in Europe also crumbled away. Save for a few pockets outside of Europe and Africa, capitalism’s real existing Other disappeared, and with it the ‘Iron Curtain’ and system competition that, for thousands of African students, had opened up rather than closed avenues of capital accumulation and agency. These avenues had included passing through different and evolving socialisms, and a skilful manoeuvring in the convergences and divergences between them.

